# Supplementary figures and images for: Identification of Genes Regulating Cell Death in Staphylococcus aureus
Source: Front Microbiol. 2019 Oct 1;10:2199. doi: 10.3389/fmicb.2019.02199 (PMC6779855; doi:10.3389/fmicb.2019.02199)

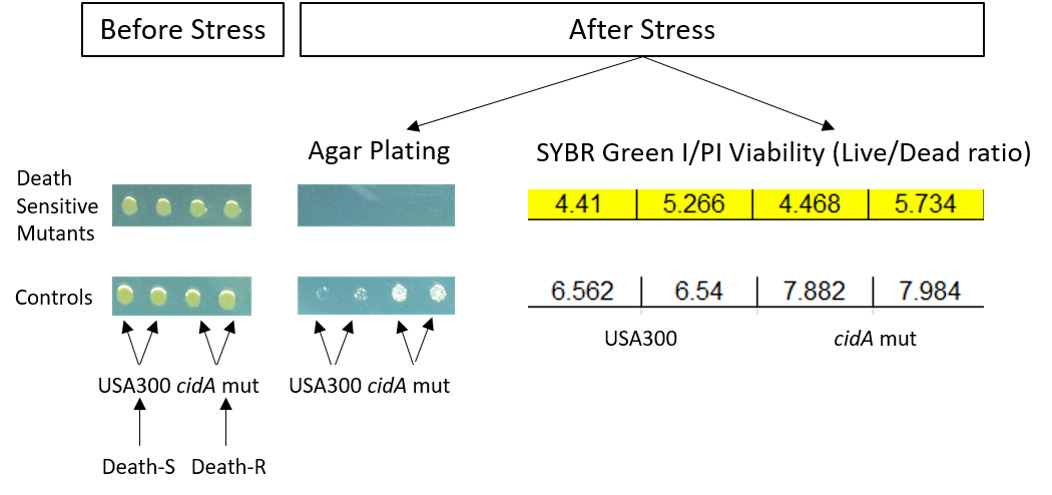

Supplement: FIGURE S1 — Comparison of agar plating and viability staining of SYBR Green I/PI. [file Image_1.jpg]
